# MetaRes-DMT-AS: A Meta-Learning Approach for Few-Shot Fault Diagnosis in Elevator Systems

**DOI:** 10.3390/s25154611

**Published:** 2025-07-25

**Authors:** Hongming Hu, Shengying Yang, Yulai Zhang, Jianfeng Wu, Liang He, Jingsheng Lei

**Affiliations:** 1 School of Information and Electronic Engineering, Zhejiang University of Science and Technology, Hangzhou 310023, China; 222208855022@zust.edu.cn (H.H.);; 2 School of Information Science and Technology, Zhejiang Shuren University, Hangzhou 310015, China; 3 Zhejiang Xinzailing Technology Co., Ltd., Hangzhou 310051, China

**Keywords:** fault diagnosis, meta-learning, Prototypical Networks, Gram Angle Field

## Abstract

Recent advancements in deep learning have spurred significant research interest in fault diagnosis for elevator systems. However, conventional approaches typically require substantial labeled datasets that are often impractical to obtain in real-world industrial environments. This limitation poses a fundamental challenge for developing robust diagnostic models capable of performing reliably under data-scarce conditions. To address this critical gap, we propose MetaRes-DMT-AS (Meta-ResNet with Dynamic Meta-Training and Adaptive Scheduling), a novel meta-learning framework for few-shot fault diagnosis. Our methodology employs Gramian Angular Fields to transform 1D raw sensor data into 2D image representations, followed by episodic task construction through stochastic sampling. During meta-training, the system acquires transferable prior knowledge through optimized parameter initialization, while an adaptive scheduling module dynamically configures support/query sets. Subsequent regularization via prototype networks ensures stable feature extraction. Comprehensive validation using the Case Western Reserve University bearing dataset and proprietary elevator acceleration data demonstrates the framework’s superiority: MetaRes-DMT-AS achieves state-of-the-art few-shot classification performance, surpassing benchmark models by 0.94–1.78% in overall accuracy. For critical few-shot fault categories—particularly emergency stops and severe vibrations—the method delivers significant accuracy improvements of 3–16% and 17–29%, respectively.

## 1. Introduction

### 1.1. Fault Prediction

Elevators are essential transportation tools that integrate electrical, mechanical, and information technologies. Property and life safety of the user may be jeopardized by any malfunction, as their daily usage frequency is exceedingly high. In modern elevator systems, stringent requirements for high availability and high safety have been set. Traditional methods relying on manual inspections and post-failure repairs can no longer meet these demands. Therefore, research and application of intelligent fault diagnosis technology have become key approaches and important breakthroughs for improving elevator operation efficiency and ensuring passenger safety [1,2].

The elevator cab operation parameters contain rich information. The kinematic state variables of the elevator carriage, specifically the instantaneous velocity v(t) and displacement s(t), can be derived through successive integration of the acceleration time series a(t). As shown in Equations (1) and (2).

(1) $$v\left(t\right)=\int_{t_{0}}^{t}a\left(\tau \right)\;d\tau +v_{0}$$

(2) $$s\left(t\right)={\;\int \int \;}_{t_{0}}^{t}a\left(\tau \right)\;d\tau^{2}+v_{0}\left(t-t_{0}\right)+s_{0}$$

where $v_{0}$ and $s_{0}$ represent initial conditions obtained from the encoder measurements [3]. The advantages of using acceleration for fault diagnosis include the fact that it only requires simple data collection, and additionally, it can effectively diagnose elevator faults. For example, triaxial accelerometers were employed by Iulian Lupea [4] to identify four health states of a helical gearbox: one normal case and three fault cases. The resulting detections were highly accurate. But there is one big problem with using deep learning to make intelligent defect diagnosis systems work. In real-life elevator situations, it is sometimes hard to obtain enough fault data, and training deep learning fault diagnostic models usually requires a lot of labeled data, which makes these methods less useful in real life. To address the issue of incomplete data, Shao et al. [5] proposed an artificial intelligence-enhanced fault prediction method that effectively handles missing information. Fault prediction methodologies are broadly categorized into mechanism-based and data-driven approaches [6]. While mechanism-based models rely on mathematical representations of physical systems, data-driven methods have gained prominence for complex systems where explicit modeling proves challenging. Deep learning has significantly advanced data-driven fault prediction, with Long Short-Term Memory (LSTM) networks [7] emerging as pivotal solutions for temporal dependency modeling. In elevator diagnostics, Liu et al. [8] demonstrated LSTM’s effectiveness in operational failure prediction, though its computational complexity and data requirements remain limiting. Subsequent hybrid architectures combining CNNs and LSTMs [9,10] improved feature extraction efficiency. Zhang et al. [10] pioneered a Wide-kernel Deep CNN (WDCNN) to enhance vibration signal processing, establishing new benchmarks for bearing diagnostics. Further innovations include WDCNN-DLSTM fusion for improved discriminative capability [11], WDCNN-GRU hybrids addressing variable-speed conditions [12], and Compact Transformers (CCTs) enabling few-shot learning [13].

Despite these advances, fundamental limitations persist for elevator fault diagnosis: significant performance degradation occurs under extreme class imbalance; inadequate generalization to rare fault categories remains problematic; and substantial data requirements are impractical for real-world deployment. As evidenced by our elevator dataset analysis (Section 3.1.2), critical faults like emergency stops exhibit severe data scarcity), rendering conventional approaches insufficient.

In addition, regarding the issue of data imbalance, Wang et al. [14] developed a self-weighted graph attention network (SW-GAT) that utilizes motor current signals to diagnose faults under severe class imbalance and introduces an inter-class adjustment loss function to enhance minority class recognition. Meanwhile, the dynamic dual-scale normalized fusion network (DSNFNet) proposed by Wang et al. [15] significantly improves the cross-domain diagnostic capability of gearboxes under variable speed conditions through graph structure modeling and subdomain distribution alignment.

### 1.2. Meta-Learning for Few-Shot Diagnosis

Meta-learning addresses these limitations through learn-to-learn paradigms [16], enabling rapid adaptation to novel tasks with minimal samples. Unlike transfer learning, which requires overlapping fault categories between source and target domains [17], meta-learning acquires transferable knowledge across diverse tasks via episodic training [18].

Recent advances demonstrate meta-learning’s efficacy in fault diagnosis: Li et al. [19] integrated meta-learning with LSTM architectures, achieving 5–12% accuracy improvements; Song et al. [20] applied meta-learning to rolling bearing fault detection; and Ma et al. [21] developed industrial-compatible meta-learning with multi-scale dilated convolution. Two predominant meta-learning frameworks have emerged: metric-based approaches—including Prototypical Networks [22], Relation Networks [23], and Matching Networks [24]—and optimization-based methods such as MAML [25], Reptile [26], and LEO [27]. Applications span medical imaging [28] and wind turbine diagnostics [29], demonstrating meta-learning’s versatility.

However, critical implementation challenges remain unresolved: manual configuration of support/query sets requires domain expertise; performance sensitivity to hyperparameter selection persists; and limited validation exists in complex electromechanical systems. As highlighted by Liu Xiu and Aldrich Chris [30], the need for human intervention in configuring shot parameters (1-shot vs 5-shot) significantly constrains practical deployment. This necessitates automated frameworks capable of self-adapting to diverse fault scenarios—the core motivation for our MetaRes-DMT-AS approach. Recent research by Kong et al. [31] has demonstrated the effectiveness of residual analysis in reducing the dependency on manual configuration for early fault diagnosis in hydraulic systems.

This research suggests a way to find elevator acceleration faults based on better meta-learning to fix these issues. This paper makes the following contributions:

(1) A better meta-learning approach for acceleration fault detection in elevators is suggested to address the problem of few-shot detection, which requires a lot of labeled data.
(2) A regularization module is added to the prototype network to solve the impact of category imbalance and improve the recognition stability of the detection method.
(3) The module dynamically adjusts the number of support sets and query sets by monitoring and adjusting the cycle interval and performance threshold, thus solving the difficulty of manual experience and manual setting of support sets and query sets.

## 2. Methods

Firstly, let us delve into the core distinctions between MetaRes-DMT-AS and existing meta-learning approaches, such as prototype networks and MAML. In contrast to prototype networks, we incorporate regularization terms by adding extra components to the loss function, thereby preventing overfitting and enhancing the model’s generalization capabilities. When compared to MAML, traditional methods rely on fixed task sampling (such as 5-way 1-shot). However, our proposed method dynamically adjusts task difficulty based on training performance; for instance, when the model learns efficiently, it automatically decreases the sample size to boost generalization. Conversely, when learning encounters obstacles, it increases the sample size to strengthen feature learning. Furthermore, we specifically focus on “hard samples” (samples with incorrect predictions) by repeatedly predicting them to enhance the model’s recognition abilities.

**Figure 1:**
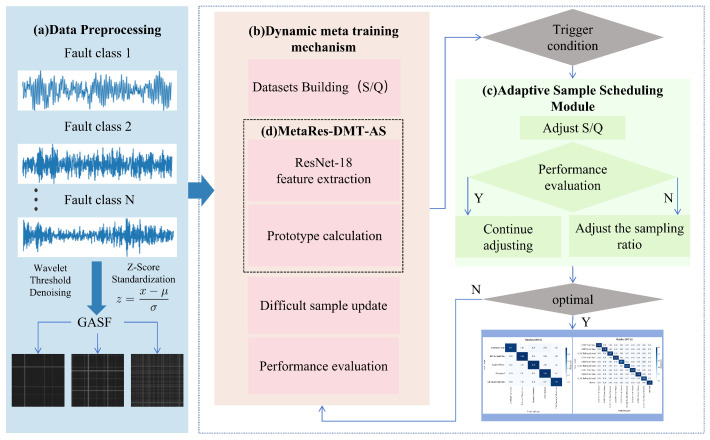
Overall flowchart of MetaRes-DMT-AS method.

Using limited data to find problems in new working conditions is like a standard few-shot classification problem, which is hard for traditional deep learning models to tackle. So, we use meta-learning theory and come up with the MetaRes-DMT-AS approach, which is presented in Figure 1. There are three steps in the whole process: (a) Data Preprocessing, (b) a dynamic meta-training mechanism, and (c) an Adaptive Sample Scheduling Module.

Firstly, we provide a brief overview of the model’s workflow. Initially, we clean the initial data and then convert one-dimensional data into two-dimensional images. We then begin constructing training tasks and randomly select five types of faults, with a minimum of one sample for each type. We monitor the performance of the model training process by recording changes in accuracy after each round of training. Based on the model’s performance (accuracy improvement or decrease), we adjust our strategy (reducing or increasing sample size) accordingly. The threshold is the number of times the performance does not improve in each cycle, which is selected based on the model’s ability. For example, if the number of times the performance does not improve reaches 3 within 15 epochs, the support set or query set will be dynamically adjusted. This threshold is 3, and the final experimental parameter setting is the best parameter selected from the results of the comparative experiment. Difficult samples refer to samples that have failed model predictions and will be prioritized for prediction in the next round until the iteration is completed. We shall go into more detail about these things below.

### 2.1. Data Preprocessing

To process the acceleration 1D sequence data in this paper, the GASF method is employed, with the following specific steps: Step 1. Assuming that the acceleration data collected by the sensor is X = x1, x2, x3,…, xn−1, xn, first normalize it through Equation (3) and scale it to [−1, 1].

(3) $${\tilde{x}}_{-1}^{i}=\frac{\left(x_{i}-max\left(X\right)\right)+\left(x_{i}-min\left(X\right)\right)}{\max(X)-\min(X)}$$

Step 2. Convert the scaled acceleration time series from step one into polar coordinates. Pay attention to two variables: the value of acceleration and its corresponding timestamp. These two variables can be represented by the angle and radius in polar coordinates, respectively. Encode on the polar coordinate system, using the cosine of the angle to encode the scaled acceleration value, which ranges between [0, π]. The corresponding timestamp should be encoded as the radius using the formula presented in Equation (4):

(4) $$\phi =\arccos\left({\tilde{x}}_{i}\right),-1\leq {\tilde{x}}_{i}\leq 1,{\tilde{x}}_{i}\in \tilde{X};r=\frac{t_{i}}{N},t_{i}\in N$$

In Step 3, the acceleration time series is transformed into polar coordinates in Step 2 to make it contain time information so GAF can be used to reconstruct the acceleration time series. In this paper, the expression of Gram Angle and Field (GASF) is expressed as Equation (5).

(5) $$\mathrm{GASF}\;=\left[\cos\left(\phi_{i}+\phi_{j}\right)\right]=\tilde{X}\prime \cdot \tilde{X}-{\left(\sqrt{I-{\tilde{X}}^{2}}\right)}^{\prime }\cdot \sqrt{I-{\tilde{X}}^{2}}$$

### 2.2. Dynamic Meta-Training Mechanism

**Figure 2:**
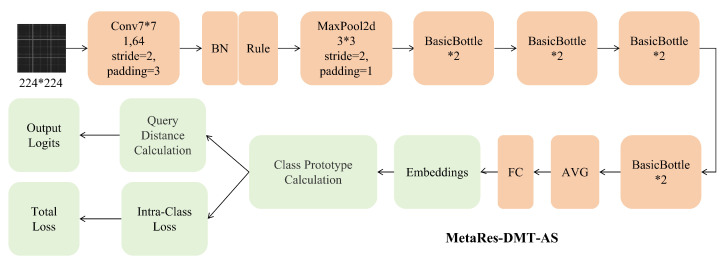
MetaRes-DMT-AS model framework diagram.

We choose ResNet-18 as the backbone of the MetaRes-DMT-AS model, as shown in Figure 2. After ResNet-18 feature extraction, the features input into the prototype network are used for prototype calculation to obtain their distance from the prototype. Additionally, we introduce intra-class distance regularization in the prototype network to enhance the quality and stability of the prototypes, as shown in Equation (6): original loss function, regularization term, and regularization coefficient.

(6) $$\tilde{J}\left(\theta \right)=J\left(\theta \right)+\lambda \cdot \Omega \left(\theta \right)$$

Finally, We use a regularization coefficient of 0.1. Equation (7) shows the number of categories, the number of samples that are supported for each category, the feature vector of the sample of the category, and the prototype of the category.

(7) $$L=L_{\mathrm{CE}}+\lambda \cdot \frac{1}{N}\sum_{c=1}^{N}\frac{1}{K}\sum_{k=1}^{K}{∥x_{k}^{c}-p_{c}∥}_{2}$$

Considering the problem of dataset classification scarcity and lack of data in industrial scenarios, the meta-dataset and test set are included under the same fault classification conditions, aiming to improve the accuracy of a few sample categories. Difficult sample mining: sampling based on difficulty, as shown in Equation (8):

(8) $$Q_{c}=\mathrm{Sample}\;n_{\mathrm{hard}}\;\mathrm{samples}\;\mathrm{from}\;H_{c}\;\cup \;\mathrm{Take}\;(n_{\mathrm{query}}\;-n_{\mathrm{hard}})\;\mathrm{from}\;\mathrm{ordinary}\;\mathrm{samples}$$

Finally, the difficult example library is updated, as shown in Equation (9):

(9) $$\mathrm{if}\;{\hat{y}}_{i}\neq y_{i}\left(\mathrm{prediction}\;\mathrm{error}\right),\mathrm{add}\;x_{i}\;\mathrm{to}\;H_{y_{i}}$$

### 2.3. Adaptive Sample Scheduling Module

The proportion of the support set to the query set in the meta-learning architecture has a direct bearing on the degree to which the model is able to generalize and how well it is able to capture information about the task at hand. This study suggests a parameter-adaptive adjustment algorithm that is based on performance monitoring. The main idea behind it is made up of the two main parts presented in Algorithm 1.
**Algorithm 1** Adaptive Sample Scheduling Module1:**procedure** AdaptiveTraining
2:    **Initialization:**
3:    θ←RandomInit()▹ Model parameters4:    H←∅▹ Hard sample repository5:    $S\leftarrow S_{\mathrm{init}},\;Q\leftarrow Q_{\mathrm{init}}$
6:    $\rho \leftarrow \frac{S}{S+Q}$▹ Initial support ratio7:    no_improve_count←0▹ Stagnation counter8:    $A_{\mathrm{best}}\leftarrow -\infty$▹ Best accuracy9:    **while not** converged **do**
10:          MiniBatchTraining(θ, *S*, *Q*)
11:           **if** epochmodT=0 **then**
12:                 $A_{\mathrm{val}}\leftarrow \mathrm{Evaluate}\left(\theta \right)$
13:                 **if**
$A_{\mathrm{val}}>A_{\mathrm{best}}$ **then**
14:                       $A_{\mathrm{best}}\leftarrow A_{\mathrm{val}}$
15:                       no_improve_count←0
16:                       H←H∪CollectHardSamples()
17:                 **else**
18:                       no_improve_count←no_improve_count+1
19:                       **if** no_improve_count≥τ **then**
20:                             $\rho \leftarrow \min(\rho +\rho_{\mathrm{step}},\rho_{\max})$
21:                             N←S+Q▹ Maintain total samples22:                             S←max(⌊ρN⌋,1)
23:                             Q←max(N−S,1)
24:                       **end if**
25:                 **end if**
26:           **end if**
27:    **end while**
28:    **return** θ
29:**end procedure**


Trigger Condition: The system monitors the performance improvement of the model at periodic intervals (adjust_interval). Specifically, after each T training round (epoch), the algorithm checks whether the no_improvement_counter (no_improve_count) has reached the preset threshold (no_improve_threshold). This counter records the number of consecutive rounds without performance improvement since the last improvement, as shown in Equation (10). t represents the current training round, T is the adjustment interval period, and is the performance stagnation tolerance threshold.

(10) $$Trigger\left(t\right)=\left\{\begin{array}{cc} 1 & \mathrm{if}\;t\;\mathrm{mod}\;T=0\;\wedge \;\mathrm{no}\_\mathrm{improve}\_\mathrm{count}\geq \tau \\ 0 & \mathrm{otherwise} \end{array}\right.$$

Parameter Adjustment Priority Strategy: The adjustment order (adjust_order) is implemented using a circular queue mechanism to achieve multi-parameter collaborative optimization. By performing modular operations (epoch // adjust_interval% len(adjust_order)), the current adjustment object is dynamically selected, ensuring equal adjustment opportunities for both support sets and query sets. For example, when adjust_order is set to [‘support’, ‘query’], the system alternates between adjusting the two parameters to avoid frequent changes in a single parameter that could disrupt training stability. Equation (11) is as follows: the parameter set P = S, Q, where p is the value of parameter p in round t; the upper and lower bounds of the parameter, respectively; and the change in accuracy.

(11) $$n_{p}^{t+1}=\left\{\begin{array}{cc} \min\left(n_{p}^{t}+1,n_{\max}^{p}\right) & \;\mathrm{if}\;\Delta_{\mathrm{acc}}\leq 0\\ \max\left(n_{p}^{t}-1,n_{\min}^{p}\right) & \;\mathrm{otherwise}\; \end{array}\right.$$

## 3. Results

This section provides a comprehensive overview of the dataset, implementation details, results, discussion, ablation studies, and confusion matrix comparison results.

### 3.1. Dataset Introduction

The proposed model was tested using two separate sets of data. The CWRU bearing fault dataset and the elevator fault acceleration dataset were collected separately and made up these datasets.

#### 3.1.1. Cwru Dataset

Common bearing fault diagnosis benchmark data is the Case Western Reserve University Bearing Fault Database (CWRU). The dataset contains bearing vibration data from different fault modes and operating conditions.

**Figure 3:**
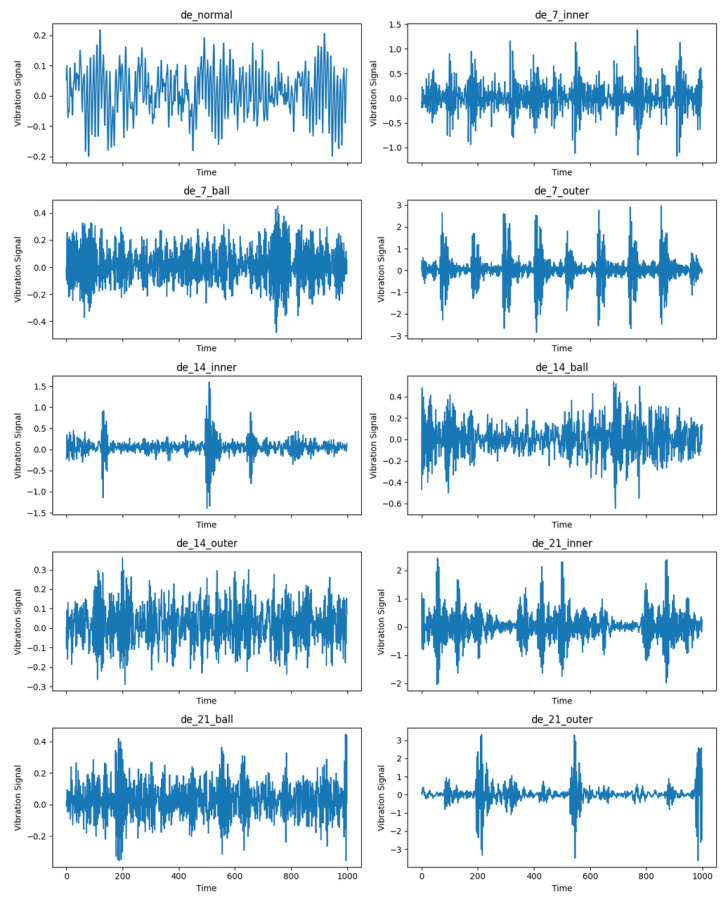
Acceleration changes in ten categories of CWRU.

We used the 12k Drive End Bearing Fault Data from this dataset for our studies. The sampling frequency was 12 kHz. There are three kinds of data in this dataset: DE drive end acceleration data, FE fan end acceleration data, and BA base acceleration data. We solely used the DE data to make the dataset, which has three kinds of faults: inner race faults, outer race faults, and rolling element faults. There are three different fault widths for each type of defect: 0.007 inches, 0.014 inches, and 0.021 inches. Each fault diameter also has data at four other speeds: 1797 rpm, 1772 rpm, 175 rpm, and 1730 rpm. Figure 3 shows the vibration acceleration variations of CWRU under ten different operating settings for the last group, which is normal data.

We divided the data into data segments of length 1000 with an overlap rate of 50%, and then processed it with the same GASF method. Finally, the image input into our proposed method was converted to 224 * 224 resolution for training. The specific data of ten types of faults are shown in Table 1.

#### 3.1.2. Elevator Fault Dataset

**Figure 4:**
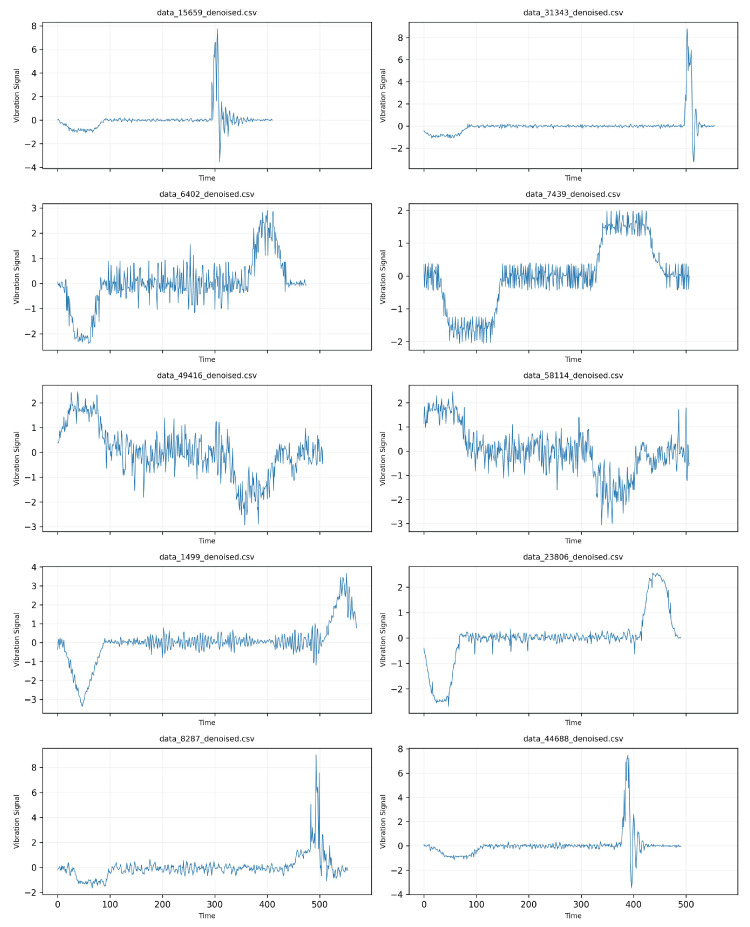
Visualization of elevator acceleration faults.

The dataset is composed of three-axis combined acceleration (mm/s^2^) of five elevator operating conditions (overspeed, reset operation, emergency stop, severe car oscillation, and normal operation) collected by the gyroscope kx023 installed on the top of the elevator car. The selected five types of faults (overspeed, reset operation, emergency stop, serious car vibration, normal operation) cover the key safety risk scenarios of the elevator system and meet the actual industrial needs (such as emergency stop and serious vibration are directly related to passenger safety). Limited by the industrial site conditions, the occurrence frequency of serious faults (such as emergency stop) is low, and it is difficult to obtain more samples of fault types. However, the selected categories are typical and engineering representative. The occurrence rate of serious faults (such as emergency stop) in industrial scenarios is naturally low, resulting in sample scarcity. This is exactly the realistic motivation of this study. In data cleaning, the incomplete fragments in 16,794 original data are removed, and the available sample size of low-frequency faults is further compressed through unified input of standardized length (400 points). Emergency stop (157 samples) and severe vibration (116 samples) are low-frequency serious faults, and the probability of natural occurrence is far lower than that of conventional operation (for example, the sample size of normal operation is about 1500), which conforms to the real data distribution of elevator operation. Figure 4 shows the visualization of acceleration of elevator fault dateset.

**Figure 5:**
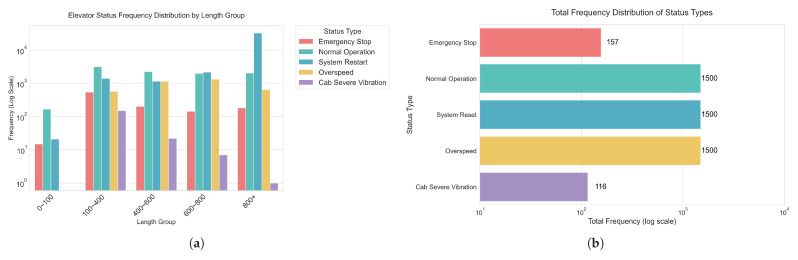
(**a**) Original data acceleration length distribution. (**b**) Number of datasets after unified 400 length.

The details are as follows: The original data includes 16,794 operation processes, some of which contain incomplete or missing data, which are identified as irrelevant information and deleted. Additionally, the duration of each operating condition varies; as shown in Figure 5a, most over-speed conditions occur between 400 and 600, most reset operations occur above 800, most emergency stops occur between 100 and 400, most severe cabin oscillations occur between 100 and 400, and most normal operations also occur between 100 and 400. Finally, the intervals corresponding to each different operating condition with the longest duration, uniformly set at 400, are processed. Insufficient intervals are interpolated using cubic splines, and excess intervals are extracted, as shown in Figure 5b, which illustrates the visualization of elevator acceleration faults.

After processing, it is found that, as shown in Figure 5b, the data of emergency stop and severe vibration of the elevator car are far less than the other three kinds of 1500 pieces each, with only 157 and 116 pieces. Finally, these data are normalized by z-scores and denoised by wavelets to meet the standard of the dataset.

Finally, as shown in Table 2, we compared STFT and GASF in one-dimensional signal conversion image algorithms and conducted comparative experiments. We found that GASF can better extract features. Especially in the Emergency Stop and Cab Severe Vibration data types, there is a difference of 6% and 13% respectively, so we uniformly choose the GASF method for signal processing.

### 3.2. Experimental Settings

We compared the suggested MetaRes-DMT-AS for fault diagnosis with five other approaches to see how well it worked: (1) WDCNN; (2) WDCNN-DLSTM; (3) WDCNN-GRU; (4) ViT; (5) CCT; (6) Prototypical Network; and (7) MAML. All of the models were tested in the same operating environment to make sure that the tests were fair and consistent. A 13th Gen Intel (R) Core (TM) i5-13400F 2.50 GHz CPU, NVIDIA GeForce GTX4060 Ti GPU, Windows 11, CUDA 12.4, and PyTorch 1.13.0 are all part of this environment.

Adam is the optimizer, and CrossEntropyLoss is the loss function in this test. The accuracy of a single defect and the overall accuracy of all categories serve as our evaluation criteria. Table 3 shows the hyperparameter settings for MetaRes-DMT-AS on CWRU. The learning rate is 0.0001, the number of iterations is 100, the number of episodes per epoch (the number of tasks created in each training cycle) is 50, the batch size is 32, and the number of support and query is 6 and 6, respectively.

The MetaRes-DMT-AS hyperparameter settings on CWRU are displayed in Table 4. The batch size is 32, the learning rate is 0.0001, there are 100 iterations, 50 episodes per epoch (the number of tasks generated in each training cycle), as well as three query and support tasks.

### 3.3. Performance Evaluation

This experiment conducted fault diagnosis on the data, utilizing eight deep learning models (MetaRes-DMT-AS, Prototypical Network, MAML, CCT, WDCNN, ViT, WDCNN-DLSTM, WDCNN-GRU) for a comparative study, all of which are set with 100 iterations of training. Table 5 and Table 6 show the inference time, memory, and accuracy of a single image in the comparative experiment.

**Figure 6:**
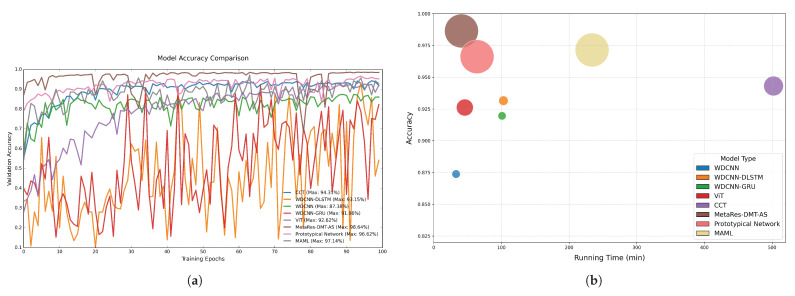
(**a**) Comparison of model iteration process. (**b**) Comparison of model parameters, accuracy, and running time.

As illustrated in Figure 6a, distinct convergence patterns and classification performance are observed across the evaluated models. The proposed MetaRes-DMT-AS exhibits superior initial performance and convergence stability, attaining the highest initial accuracy (97.12%) and progressively improving to 98.64% during training. In contrast, CCT and WDCNN demonstrate suboptimal initial accuracies (95.34% and 93.27%, respectively) and exhibit performance saturation, achieving final accuracies of 94.31% and 87.38%. The ViT model displays unique convergence behavior: despite a lower initial accuracy (89.45%), it gradually improves to 92.62% through iterative optimization, matching CCT’s performance level. Notably, the variants models of WDCNNts (WDCNN-DLSTM and WDCNN-GRU) show pronounced instability with oscillatory convergence, reaching peak accuracies of 93.15% and 91.96%. The Prototypical Network achieves the second-highest initial accuracy (following MetaRes-DMT-AS) and maintains consistent stability throughout training, ultimately securing third-place overall performance (96.62%). Although MAML commenced with lower initial accuracy and exhibited instability, it attained a final accuracy of 97.14%, surpassed only by MetaRes-DMT-AS.

Analysis of computational efficiency and model complexity (Figure 6b) reveals that MetaRes-DMT-AS achieves competitive iteration times (83 min) despite its substantial parameter count (11.18 M), indicating exceptional computational efficiency. ViT balances compact parameterization (2.76 M) with rapid execution (46 min). The WDCNN series excels in parameter efficiency (0.64–0.86 M) and computational speed (<1.10 min/iteration), though their accuracy remains substantially inferior to advanced models. CCT demonstrates notably poor computational efficiency, requiring 502 min while delivering only moderate accuracy (94.31%). The Prototypical Network and MAML exhibit similar parameter complexity (11.17 M) to MetaRes-DMT-AS, yet divergent performance profiles emerge: the Prototypical Network completes training in 64 min (96.62% accuracy), while MAML requires 230 min to achieve 97.14% accuracy.

**Figure 7:**
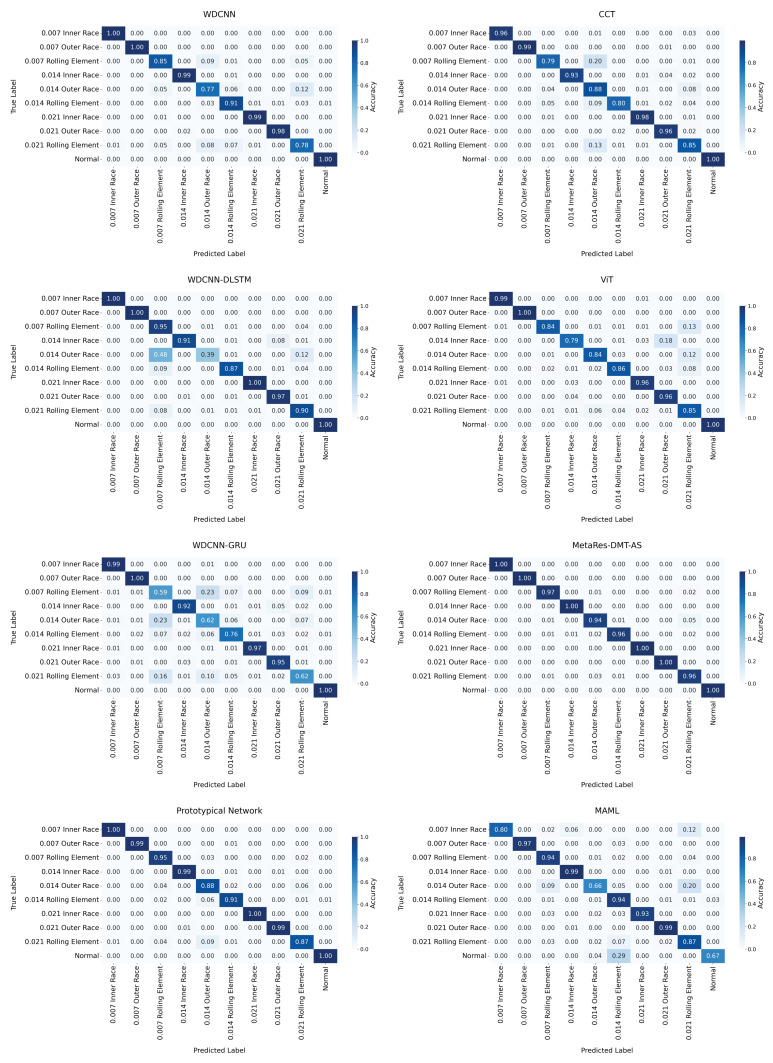
Comparison of confusion matrices of various models in CWRU.

To rigorously evaluate classification performance on challenging samples, this study conducted fine-grained recognition analysis using confusion matrices (Figure 7). The MetaRes-DMT-AS model demonstrated exceptional robustness, achieving perfect 100% recognition in six of ten fault categories while maintaining a minimum recognition rate of 94% for 0.014 outer ring failure. Comparative assessment revealed significant performance variations across benchmark models. The CCT model attained 100% recognition in three categories but exhibited substantial degradation to 85% for 0.007 rolling element, 77% for 0.014 outer ring, and 78% for 0.021 rolling element failures. WDCNN-DLSTM achieved moderate performance (79–88%) on similar challenging samples, while ViT displayed bipolar characteristics with over 95% recognition in five categories but approximately 80% in five others. WDCNN-GRU suffered severe class imbalance, yielding only 39% recognition for 0.014 outer ring. The baseline WDCNN model demonstrated critically low recognition below 65% across multiple fault categories, including 0.007 rolling element, 0.014 outer ring, and 0.021 rolling element. The Prototypical Network achieved high accuracy in most categories but declined to 88% for 0.014 outer ring and 87% for 0.021 rolling element, with intermediate performance of 95% and 91% on 0.007 rolling element and 0.014 rolling element, respectively. MAML exhibited significant instability, yielding suboptimal recognition rates of 66% and 67% for 0.014 outer ring and normal categories, and merely 80% and 87% for 0.007 inner race and 0.021 rolling element failures.

**Figure 8:**
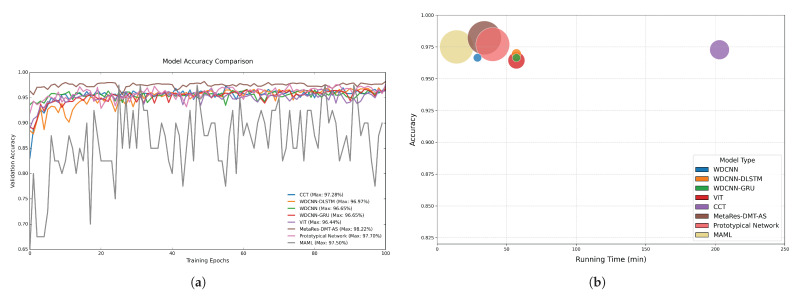
(**a**) Comparison of model iteration process. (**b**) Comparison of running time, accuracy, and number of model parameters.

In the elevator fault dataset experiment, a total of eight models were compared, all using 100 iterations. The accuracy of the comparison experiment is shown in Table 7:

As depicted in Figure 8a, distinct convergence patterns emerge during model training: The proposed MetaRes-DMT-AS achieves optimal initial recognition performance (97.85%) and steadily converges to 98.22% accuracy. In contrast, WDCNN demonstrates suboptimal initial performance (96.12%) with noticeable learning stagnation, plateauing at 96.65%. ViT, WDCNN-GRU, and WDCNN-DLSTM exhibit similar convergence trajectories, starting at 90.3 ± 0.8% mean accuracy and improving to 96.44%, 96.65%, and 96.97%, respectively, through iterative optimization. Notably, CCT displays unique anti-overfitting characteristics, progressing from 82.14% initial accuracy to 97.28% final performance. The Prototypical Network maintains exceptional stability on the elevator fault dataset, ranking third in initial accuracy and achieving 97.70% final accuracy. MAML exhibits significant instability with continuous fluctuations, yet ultimately reaches 97.50% accuracy. These results demonstrate meta-learning’s consistent advantage for few-shot learning, with all three meta-learning methods securing top-three positions across both sensor datasets.

Computational efficiency analysis (Figure 8b) reveals that MetaRes-DMT-AS maintains excellent time efficiency (34 min) despite larger parameter scale (11.18 M), validating its architectural optimization. WDCNN achieves optimal resource efficiency with streamlined parameters (0.66 M) and minimal iteration time (29 min), though exhibiting a 1.57% accuracy gap versus advanced models. the variants models of WDCNN (WDCNN-DLSTM: 0.86 M/57 min, WDCNN-GRU: 0.64 M/57 min) and ViT (2.76 M/34 min) form an intermediate performance cluster with <0.53% accuracy variation.CCT shows significant efficiency bottlenecks, requiring the highest iteration time (203 min) for a marginal accuracy advantage (97.28%). The Prototypical Network and MAML exhibit comparable parameter complexity, with the Prototypical Network achieving 97.70% in 40 min and MAML reaching 97.50% in merely 14 min.

**Figure 9:**
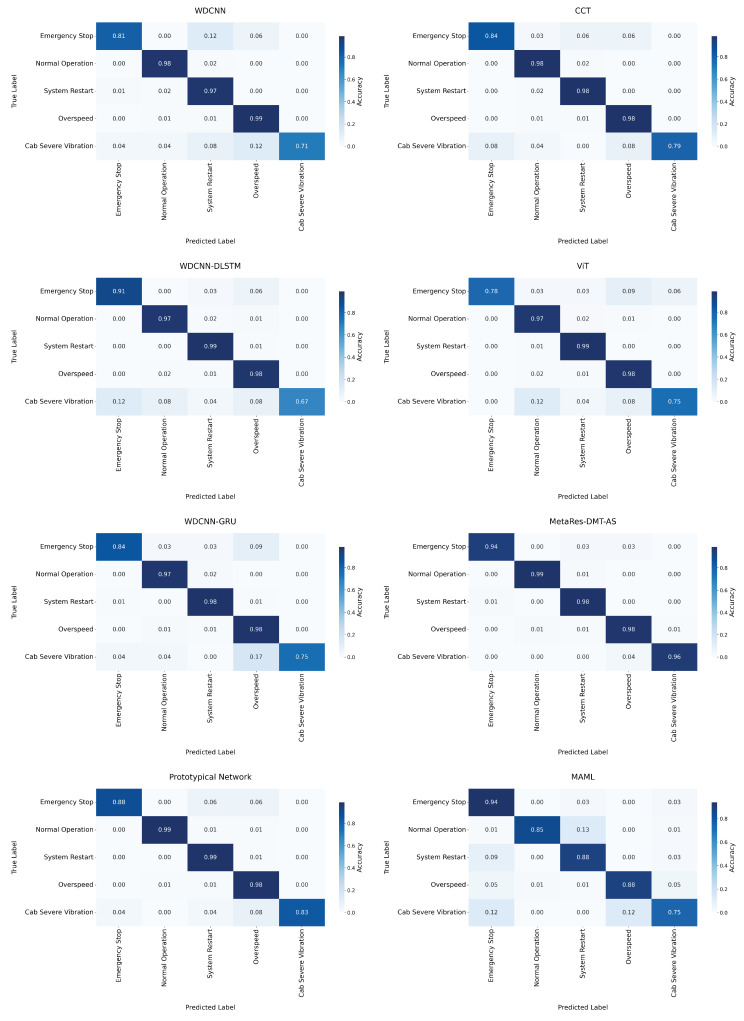
Comparison of confusion matrices of various models in the elevator fault dataset.

To evaluate model robustness in few-shot scenarios, this study performed fine-grained analysis of two critical anomalies—emergency stop and cab severe vibration—using confusion matrices (Figure 9). The proposed MetaRes-DMT-AS maintained robust recognition (98.2 ± 0.6%) for regular samples while significantly outperforming benchmark models in few-shot categories, achieving 94% for emergency stop and 96% for cab severe vibration. Comparative analysis revealed substantial performance variations across models. CCT, WDCNN-GRU, and ViT demonstrated strong performance (>95%) across diverse categories but exhibited sensitivity to data distribution, declining to 75–84% for few-shot anomalies. WDCNN-DLSTM showed significant imbalance with 91% recognition for emergency stop but plummeting to 67% for cab severe vibration, indicating limitations in temporal feature fusion. The baseline WDCNN model demonstrated substantially lower performance in both few-shot tasks (81% and 71%), underscoring the limitations of shallow architectures for complex pattern learning. The Prototypical Network yielded 88% and 83% recognition for emergency stop and cab severe vibration, respectively. MAML exhibited inconsistent performance: while achieving 94% for emergency stop, it attained only 85% for normal operation, 88% for system restart, 88% for overspeed, and 75% for cab severe vibration.

### 3.4. Ablation Study

To assess the effect of distinct elements or characteristics on overall performance, ablation experiments are utilized. Ablation experiments remove specific parts of the model step by step and analyze how these changes affect model performance. The ablation experiments we designed are shown in Table 8. To explore the effects of ablation experiments, classification rates reaching 100% for certain categories are not listed in the table below.

As shown in Table 8, the MetaRes-DMT-AS model performs optimally in classifying categories with fewer samples, indicating that the effective combination of adaptive methods based on meta-learning and regularization of prototype networks can significantly enhance model performance. After removing any module, the model’s performance declines; removing the prototype network (Siamese Networks) results in an accuracy drop of 0.21%, while the 0.014 outer race and 0.021 rolling element decrease by 1% and 3%, respectively. Removing the meta-learning module leads to an accuracy drop of 1.03%, with performance decreasing by 2%, 2%, 2%, and 6% across different categories. When both Siamese Networks and meta-learning are removed, the accuracy drops by 1.28%, with performance decreasing by 2%,3%,4%, and 9% across different categories. These results indicate that Siamese Networks and meta-learning play a crucial role in the model on this dataset.

As shown in Table 9, the MetaRes-DMT-AS model performs optimally in classifying few-shot categories, indicating that the effective combination of meta-learning-based adaptive methods and regularization of the prototype network can significantly enhance model performance. After removing any module, the model’s performance declines; removing the prototype network (Siamese Networks) results in an accuracy drop of 0.21%, with few-shot categories (emergency stop and severe cabin oscillation) decreasing by 10% and 8%, respectively. Removing the meta-learning module leads to an accuracy drop of 0.31%, with few-shot categories decreasing by 6% and 13%. If both Siamese Networks and meta-learning are removed, the accuracy drops by 0.63%, while the number of samples decreases by 10% and 13%, respectively. The above results show that Siamese Networks and meta-learning modules also played a key role in our elevator fault dataset.

Specifically, the meta-learning module helps address the imbalance issue of few-shot sizes, while the Siamese Networks module enhances feature extraction and model stability. Therefore, our proposed model, by integrating the advantages of both the Siamese Networks and meta-learning modules, can effectively extract features from few-shot data that were previously difficult to obtain, significantly improving fault classification performance in scenarios with limited samples.

## 4. Conclusions

In this paper, we propose a method called MetaRes-DMT-AS to address the problem of limited data availability in elevator fault diagnosis under complex operational conditions. A technique that combines metric learning tasks with meta-learning is suggested. By using existing bearing fault data and small amounts of elevator fault data for analysis, this method creates two experiments. The comparison results show that the improved meta-learning approach for finding elevator acceleration faults is different from the standard meta-learning approach, which needs specific datasets and processing. It can support the support set and query set in a way that adapts to the situation, making the model work better and better at recognizing challenging samples. We will keep researching intelligent fault diagnosis using a small number of samples under meta-learning in the future. On the one hand, this entails investigating hyperparameter optimization in meta-learning, including learnable learning rate selection; on the other hand, it entails strategies to minimize the number of meta-learning parameters. In addition, there is strong noise interference in the real elevator environment, and we need to further enhance the model’s ability to resist noise. Finally, considering practical applications, deploying on edge devices requires strong real-time performance.

## Figures and Tables

**Table 1:** Details of CWRU bearing dataset.

Data Type	Data Size
0.007 rolling element	969
0.007 inner race	970
0.007 outer race	970
0.014 rolling element	970
0.014 inner race	968
0.014 outer race	969
0.021 rolling element	970
0.021 inner race	969
0.021 outer race	970
normal	3388

**Table 2:** Comparison table of one-dimensional signal conversion image algorithms.

Algorithms	Emergency Stop	Normal Operation	System Restart	Overspeed	Cab Severe Vibration
STFT	88	99	99	98	83
GASF	**94**	99	98	98	**96**

**Table 3:** Hyperparameter settings of MetaRes-DMT-AS on CWRU.

Hyperparameters	Value
Learning rate	0.001
Epochs	100
Episodes per epoch	50
Batch size	32
Support	6
Query	6

**Table 4:** Hyperparameter settings of MetaRes-DMT-AS on elevator fault dataset.

Hyperparameters	Value
Learning rate	0.001
Epochs	100
Episodes per epoch	50
Batch size	32
Support	3
Query	3

**Table 5:** Single Image Inference Performance Comparison (Sorted by Inference Time).

Model	Inference Time (ms)	Peak Memory (MB)
WDCNN	2.10	2.00
MAML	2.83	2.00
CCT	3.61	5.99
Prototypical Network	4.85	6.00
MetaRes-DMT-AS	4.97	6.00
Vision Transformer (ViT)	6.01	2.00
WDCNN-GRU	47.31	2.00
WDCNN-DLSTM	48.38	2.00

**Table 6:** Comparison experiment of MetaRes-DMT-AS on CWRU.Bold is our model and accuracy.

Model	Accuracy/%
CCT	94.31
WDCNN	87.38
ViT	92.62
WDCNN-DLSTM	93.15
WDCNN-GRU	91.96
Prototypical Network	96.62
MAML	97.14
**MetaRes-DMT-AS**	**98.64**

**Table 7:** Comparison experiment of MetaRes-DMT-AS on elevator fault dataset. Bold is our model and accuracy.

Model	Accuracy/%
CCT	97.28
WDCNN	96.65
ViT	96.44
WDCNN-DLSTM	96.97
WDCNN-GRU	96.65
Prototypical Network	97.70
MAML	97.50
**MetaRes-DMT-AS**	**98.22**

**Table 8:** CWRU ablation experiment (Adding a check mark in the table indicates the use of the module, and bolding indicates optimal accuracy).

Method Configuration		Detection Accuracy (%)				
**Prototypical Networks**	**Adaptive Sampling**	**Overall**	**0.014 Inner Race**	**0.014 Outer Race**	**0.014 Rolling Element**	**0.021 Rolling Element**
		97.36	98	91	92	87
	✓	98.43	100	93	96	93
✓		97.61	98	92	94	90
✓	✓	**98.64**	**100**	**94**	**96**	**96**

**Table 9:** Elevator fault dataset ablation experiment (Adding a check mark in the table indicates the use of the module, and bolding indicates optimal accuracy).

Method Configuration		Detection Accuracy (%)					
**Prototypical Networks**	**Adaptive Sampling**	**Overall**	**Emergency Stop**	**Normal Operation**	**System Restart**	**Overspeed**	**Cab Severe Vibration**
		97.59	84	99	97	99	83
✓		98.01	84	99	99	99	88
	✓	97.91	88	99	99	99	83
✓	✓	**98.22**	**94**	**99**	98	98	**96**

## Data Availability

The study’s data are available upon request. The study’s code will be available on https://github.com/mango0423/MetaRes-DMT-AS-A-Meta-Learning-Approach-for-Few-Shot-Fault-Diagnosis-in-Elevator-Systems (accessed on 20 July 2025).
